# Chinese-Style Decentralization and Health System Reform

**DOI:** 10.1371/journal.pmed.1001337

**Published:** 2012-11-06

**Authors:** David Hipgrave, Sufang Guo, Yan Mu, Yan Guo, Fei Yan, Robert Scherpbier, Hana Brixi

**Affiliations:** 1 Nossal Institute for Global Health, University of Melbourne, Melbourne, Australia; 2 UNICEF Office for China, Beijing, People's Republic of China; 3 Department of Health Policy and Administration, School of Public Health, Peking University, Beijing, People's Republic of China; 4 Department of Health Statistics and Social Medicine, Fudan University School of Public Health, Shanghai, People's Republic of China; 5 The World Bank, Washington, D.C., United States of America

## Abstract

David Hipgrave and colleagues discuss health system reform in China and argue that parallel reforms in governance, financing, and accountability are also needed to ensure health equity.

**Summary Points**

- China's health system reform (HSR) recently entered a second phase—the State Council and a multi-agency steering committee announced many important new initiatives
- However, China's social sectors are heavily decentralized—the Ministry of Health has limited influence on the detailed design and implementation of the HSR at sub-national level
- Sub-national leaders in China are mostly driven by economic progress and revenue generation; health is generally regarded as consuming, not generating revenue
- A new health management information system and performance-based management of health staff may enhance oversight
- However, parallel reforms in governance, financing, and accountability are also needed in China, to ensure achievement of the equity objective of its HSR

## A Milestone Is Reached

April 2012 marked the end of the three years China set for implementation of the first phase of its health system reform (HSR), and several recent reports assessed progress in this massive undertaking [1]–[3]. Two of them identify impressive achievements in health insurance coverage, infrastructure development, and uptake of services [1],[3], but all three reports draw attention to slow progress in several areas: quality of services at the community level, persisting financial risk for individuals, and the almost complete lack of independent tracking of progress in health outcomes. The reports also recommend an increased focus on burgeoning rates of non-communicable diseases [2]–[4]. Government-commissioned external reviews also highlighted these and other issues, underscoring related high-level awareness. However, none of the reports attempted to place China's HSR in the context of the nation's evolving political economy, or to predict its influence on achievement of the major HSR objective—equitable and affordable access to quality health services.

## Moving Forward without Looking Back

The rationale for, components of, and intrinsic challenges to HSR in China are summarised in Box 1. Strengthening grassroots health care and ensuring universal access to basic primary and public health services are among its five pillars, and echo the outgoing national leaders' overarching focus on social harmony through improving social services and poverty reduction [5]. Equity in access to health services is indeed improving [1], and even before the recent HSR reviews, related national initiatives were being announced with impressive scale and ambition.

**Box 1. Health System Reform (HSR) in China**

- **The rationale for HSR in China**
  1. Marketisation and poor funding of China's health sector had made health care unaffordable for many.
  2. Although the majority of health facilities are owned by government, in 2001 around 60% of total health expenditure was out of pocket. Insurance coverage was very low.
  3. Health care is mostly fee-for-service, but fees and salaries are set artificially low. Drug sales and tests provide alternative income for facilities and providers.
  4. Hospitals are managed as profit-making enterprises, rather than for the public good.
  5. Public health and information systems were particularly weak, as seen during the SARS crisis.
- **China's plans to achieve universal coverage by 2020**
  1. China's HSR comprises five pillars developed by a national multi-agency committee:
    1. Expanding the coverage of and benefit provided by subsidised health insurance;
    2. A drug reform scheme with zero mark-up on listed essential medicines;
    3. Improving access to and the quality of primary health care;
    4. Basic public health screening and management at community level for all, and
    5. Public hospital reform.
  2. A number of targeted programs have also been developed by the Ministry of Health.
  3. The target date for completing the reform is 2020.
- **Intrinsic challenges to China's HSR**
  1. A generation of health providers and local governments regard health care as essentially a private business, and will resist efforts to control fee and other income.
  2. The majority of health providers are underqualified, especially in rural areas; training new providers and overcoming a heavy urban bias in their health-worker distribution will take years [31]–[33].
  3. With the exception of a radically improved notifiable disease reporting system [34], China's HMIS remains weak.
  4. Although increasing, funding of the health sector is heavily decentralized and regressive [5],[18].
  5. Local health authorities are encouraged to further develop, fund, and pilot the HSR components, resulting in inconsistent progress and output.

In a July 2011 “Guidance,” the State Council announced new roles for the grassroots providers originally called “barefoot doctors,” committing them to a wide range of tasks [6]. By 2020, China's village doctors should be providing standardised primary care (following new clinical guidelines); implementing public health programs; undertaking disease surveillance; conducting community education; participating in health financing schemes; and maintaining individual e-health dossiers. A second, related initiative is a major new health management information system (HMIS) to be introduced over 2011–2015 and linked to China's civil registration MIS. Allocated a budget of 22 billion renminbi (RMB) in 2011 (almost US$3.5 billion), this system will potentially feed real-time information upwards to decision makers and health resource managers, and also enable top-down monitoring of health facilities and providers, theoretically even at the village level. Although the data will not be collected independently, this system may reduce the current information gap and also assist introduction of a third initiative, a performance management and incentive program intended to assess and potentially reward the activity of health workers and facilities at all levels, facilitating staff rotation and encouraging health professionals to work in rural areas.

These new initiatives anticipated and responded to the gaps highlighted in recent reviews and are welcome to those familiar with community health in rural China. In 2010, only 14.2% of China's village doctors were appropriately qualified [7]; although training of government health staff is improving [8], professional standard-setting in China is weak [9], particularly at the grassroots level [5]. The official engagement and monitoring of village doctors in a national system is an optimistic but positive development, and should improve public confidence in grassroots services. Despite their legendary role in China's long-established three-tier health network (village, township, and county), these cadres have never been formally co-funded by the national government.

## National Commitment to HSR Remains Strong

Following these initiatives, in March 2012, China's State Council announced a new phase of HSR, underscoring the ongoing commitment of the highest echelons of government [10]. The new four-year plan (2012–2015) reiterates the goal of universal access to basic health services by 2020, and specifically refers to supply constraints amid growing and diversifying health needs. It focuses on many of the areas recommended in the recent reviews, including: expanding insurance benefits and unifying China's several health insurance schemes; encouraging the development of commercial insurance, the private sector (targeted to manage 20% of health services by 2015), capitation and other payment reforms to separate doctors from the financial management of hospitals; health-worker performance-based funding; family general practice; expanding community and public health services; and consolidation and regulation of drug production, prescription, and pricing. The plan is encouragingly specific but not prescriptive on health sector strategy. However, it remains vague on accountability and local spending responsibilities, stipulating only that government health spending gradually increase as a proportion of total recurrent government expenditure. This vagueness hints at the major problem for the equity objective of China's HSR.

## Local Government Support for China's HSR Is Imperative

China's HSR is a national process but its implementation, funding, and evaluation exemplify the hazards of the nation's style of decentralization. Chinese federalism involves three major principles: hierarchical administrative sub-contracting (the devolution of responsibility for implementation of national policy to progressively lower levels) and territory management; inter-governmental financial transfers; and vertical competition for promotion in the public sector [5],[11]. In theory, decentralizing the financing and administration of the health sector can enhance the quality, equity, and responsiveness of local services, but this assumes prioritisation by local authorities and adequate vertical and horizontal accountability and governance [12]. In China, local government remains largely accountable to higher-level authorities, not the local population, and economic, not social development is its primary objective [11]. Moreover, since tax reforms in the mid-1990s, the funding of social services in China involves centralised revenue collection and complex fiscal transfers, but decentralized responsibility for the majority of sectoral allocations and expenditure [5]. As total revenue volumes strongly favour richer provinces and a large proportion of local government revenue is off-budget (and able to be allocated at local authorities' discretion), there is effectively a nested financial hierarchy, with deeply unequal, regressive capacity of local governments to fund social services [5],[13]–[15].

While there are promising moves to make local government more accountable to the well-being of the public (such as introduction of “green gross domestic product” measurement, and independent surveys of public opinion on local government performance in some provinces), the main motivation for sub-national authorities, including for their career trajectory, remains economic development and revenue generation [11]. Moreover, while some sectors of China's economy (banking, communications, etc.) are carefully regulated and monitored from above, the health sector is largely organised and monitored at the local level. Indeed there is no equivalent social sector governance hierarchy and it is too costly for China's undermanned central government to independently monitor and evaluate sub-national health performance [5],[11]. These circumstances explain the limited ability of national health officials to ensure reforms are pursued at grassroots level, and underscore the potential importance of initiatives like the new HMIS and performance evaluation system.

## Weak Incentives to Prioritise Health at the Sub-National Level

In recognition of the need for more support, over the last decade there were serious attempts to increase national funding for the health sector [3],[8], but it was recognised long ago that effective, equity-enhancing reform of China's health sector required parallel reforms in sub-national financing and governance [14]. Multiple state-level agencies collectively developed the five HSR themes and providing partial funding, but its detailed design, majority funding, implementation, and monitoring rely heavily on sub-national authorities [1],[3],[5],[16]–[18]. Of the RMB850 billion three-year allocation to HSR in 2009, the central government only funded 331.8 billion [19], and substantial input by local governments is again expected for the initiatives announced in 2011 [20] and 2012 [10]. For example, payment for the planned elevation of village doctors' roles and responsibilities will derive from at least three funding streams (nationally funded vertical programs; local compensation for income lost with drug reforms; and payments from the rural cooperative medical [insurance] scheme [RCMS]) [6],[7]. Oversight of these complex and fragmented payment arrangements and provision of additional funding will rely on county-level authorities [6] whose accountability for this national initiative is to local government [5],[11].

However, many sub-national governments in China view health and other public goods as consuming resources rather than as an investment in the future [2],[21]. Moreover, while local government input to supply-side initiatives and the RCMS has been critical [3],[8], broader local commitment to national health priorities requires not only money but also stewardship of health providers and coordination of other sectors not mentioned in the HSR. Again, this oversight costs money and may be viewed as impeding local economic development. Many examples, especially pertaining to food safety, drug production, and the health risks of local industries [22] imply low levels of sub-national government commitment to health, but apart from isolated crises (tainted-milk, fake vaccines, etc.), there remain few significant incentives for local government leaders to prioritise the health sector.

Moreover, recent analyses have characterised public financing of the health sector between and within China's provinces as regressive [5],[18],[23], noting that insurance schemes [24],[25] and broader health resource allocation across urban-rural and regional boundaries ignore community needs [3]. Despite increased national allocations to health (from RMB258 billion in 2007, to RMB569 billion in 2010), the sourcing and detailed allocation of the majority of public funding for the health sector occurs at sub-national level [3],[5]. In striving for economic development, China's poorer provinces and counties neglect co-funding of health despite national priorities and earmarked allocations [15],[17],[18]. Furthermore, although clearer guidance from national level would probably improve the success of certain reforms, it is not forthcoming. For example, responsibility for protecting consumers from high health costs through insurance and hospital management reform has also been largely assigned to county-level authorities [8]. Many and varied payment schemes have arisen [26],[27], threatening the recommended harmonisation of health care payment, financing, and insurance across territorial boundaries. This harmonisation accrues importance as China's population becomes increasingly mobile and is a critical element of the HSR equity objective.

## In Addition, More and Independent Information Is Needed

Another characteristic of Chinese-style federalism is that reporting of most areas of China's social and economic progress relies on locally generated administrative data, the accountability of which is mostly horizontal (to local government), not vertical (upwards within the sectors), with major implications. For example, the national annual birth cohort reported by county level authorities totals around 13 million (meeting local family planning commitments), but national authorities responsible for communicable disease control through vaccination report a cohort of >16 million. Despite recommendations [28], engagement of the public in planning, monitoring, and evaluation of the health sector is very limited in China. Monitoring the equity objective of China's HSR thus relies on evidence of limited reliability; reporting of progress is largely output-based or describes numeric improvements emanating from specific, nationally designed initiatives [1], often lacking denominators [2],[3],[29] or on which the majority of progress was made before 2009 [1],[3]. Locally representative, population-based surveys on health outcomes are uncommon in China and almost never independent, so the disaggregated impact of the HSR and local initiatives on health will remain unknown except at relatively crude (regional and urban-rural) levels [1],[30]. This lack of data increases the risk that new national allocations may not substantively impact health challenges except on a few flagship indicators (such as insurance and service uptake and hospital delivery [1]), and may even have major economic implications if predictions on the cost of China's non-communicable disease burden are correct [2],[4]. In this context, quality implementation of the new HMIS, albeit another national initiative reliant on local funding, will be a critical element of assessing future progress. This is mentioned in the new plan [10], but in general the national focus on monitoring and evaluation of the HSR remains weak, reflecting the position of the health sector in China's governance hierarchy.

## Conclusion

Pursuing HSR in China has the potential to improve equity by bringing standardised primary care and public health to those whose health status implies they need it most [18]. However, it will only succeed if sub-national governments are accountable for related public resource allocation, and if vertical monitoring of the quality, equity, efficiency, and effectiveness of the health sector is improved. In addition to HSR, reforms are also needed to align the responsibilities, capacity, and monitoring of all sub-national authorities with health-related national policies, standards, and regulations, and to improve their accountability to the population. These additions go beyond the influence of the Ministry of Health, but accord with the evident priority assigned to HSR by multiple national authorities [21], the benefits to other sectors [4], and the increasing focus of the state on evaluating local government's commitment to public welfare [11].
